# Which Anthropometric Indicators Identify a Pregnant Woman as Acutely Malnourished and Predict Adverse Birth Outcomes in the Humanitarian Context?

**DOI:** 10.1371/currents.dis.54a8b618c1bc031ea140e3f2934599c8

**Published:** 2013-06-07

**Authors:** Mija-tesse Ververs, Annick Antierens, Anita Sackl, Nelly Staderini, Valerie Captier

**Affiliations:** Medical Department, Médecins Sans Frontières, Geneva, Switzerland; Medical Department, Médecins Sans Frontières, Geneva, Switzerland; Medical Department, Médecins Sans Frontières, Vienna, Austria; Medical Department, Médecins Sans Frontières, Geneva, Switzerland; Medical Department, Médecins Sans Frontières, Geneva, Switzerland

## Abstract

Currently there is no consensus on how to identify pregnant women as acutely malnourished and when to enroll them in nutritional programmes. Médecins Sans Frontières Switzerland undertook a literature review with the purpose of determining values of anthropometric indicators for acute malnutrition that are associated with adverse birth outcomes (such as low birth weight (LBW)), pre-term birth and intra-uterine growth retardation (IUGR). A literature search in PUBMED was done covering 1 January 1995 to 12 September 2012 with the key terms maternal anthropometry and pregnancy. The review focused on the humanitarian context. Mid-upper-arm circumference (MUAC) was identified as the preferential indicator of choice because of its relatively strong association with LBW, narrow range of cut-off values, simplicity of measurement (important in humanitarian settings) and it does not require prior knowledge of gestational age. The MUAC values below which most adverse effects were identified were <22 and <23 cm. A conservative cut-off of <23 cm is recommended to include most pregnant women at risk of LBW for their infants in the African and Asian contexts.

## Background

Acute malnutrition is particularly important in humanitarian emergencies where sudden change of food availability or high disease burden can cause this form of malnutrition, and consequently excessive mortality in adults^1^^,^ 2. There is little information available to identify and treat acute malnutrition among pregnant women (PW)^3^. Yet, the impact of acute malnutrition on PW is substantial and maternal malnutrition is a major cause of low birth weight (LBW)^4^^,^ 5.

Currently there is neither consensus on which anthropometric measurement should be used to identify acute malnutrition during pregnancy nor which cut-off value should be used. In emergencies or protracted crises PW are included in nutritional programmes, most frequently supplementary feeding programmes (SFP); criteria for inclusion vary.

Some programmes use the normal body mass index (BMI) cut-off value of 18.5 kg/m^2^ for adult women, assuming it is applicable for PW 6. Mid-upper arm circumference (MUAC) is often used too, but no universal cut-off points have been identified^7^. Various national nutritional protocols use the following MUAC cut-off values for inclusion of PW into SFPs: MUAC <18.5 cm (Zimbabwe 2008), <21.0 cm (Burkina Faso, Burundi 2002, DRC 2008, Guinea 2005, Madagascar 2007, Malawi 2007, Mali 2007, Niger 2006, Senegal 2008), <22.0 cm (Mozambique 2008), <22.5 cm (Zambia 2009)^7^, <23.0 cm (Indonesia 1996)^8^ and ≤23 cm (Sri Lanka 2006)^9^. According to the SPHERE Guidelines^10^, MUAC may be used as a screening tool for PW, e.g. as a criterion for entry into a feeding programme. The guidelines state that cut-off points for risk vary by country and range from 21 cm to 23 cm. SPHERE suggests <21 cm as an appropriate cut-off for selec­tion of PW at risk for growth retardation during emergencies. Some nutritional protocols enroll PW based on gestational age (mostly only in the third trimester) regardless of any anthropometric measurement^7^.

Different sections of Médecins Sans Frontiéres (MSF) are currently using MUAC <18.5 or <21.0 cm to include PW in SPFs. The United Nations High Commissioner for Refugees 11 recommends <23 cm but states also to use <21 cm, depending on the proportions of women falling under each category of MUAC and available resources^11^.

MSF Switzerland undertook a literature review (September-October 2012) with the purpose of determining values of anthropometric indicators for acute malnutrition that are associated with adverse birth outcomes. The study examined currently used indicators, such as MUAC and BMI, but also looked at other potentially important indicators, such as maternal weight for gestational age, maternal weight gain, and maternal height. The adverse birth outcomes that were studied were LBW, intra-uterine growth retardation (IUGR) and pre-term birth (PTB) as they are strongly related to infant survival 12. Though maternal mortality was regarded as an important outcome to investigate, there were insufficient studies that examined maternal anthropometry and the relation to maternal mortality. Thus, it was not included in this study.

## Methods

A literature search in PUBMED was done covering 1 January 1995 to 12 September 2012 with the key terms maternal anthropometry and pregnancy (only human studies in English were selected with an abstract and/or full text). The year 1995 was chosen as a start as it was the year that the WHO Collaborative study on maternal anthropometry and pregnancy outcomes was published, and it is regarded as a milestone publication on this topic^13^^,^ 14. The search provided 6,697 records which were subsequently narrowed down to 4,000 records when publications were filtered excluding studies with specific drugs or hormones, diseases, environmental exposure, substance abuse, triplets, twins, in-vitro fertilisation, obesity, cigarette smoking, and anaemia. The search was further limited to studies that reported on one or more of the selected anthropometric indicators (MUAC, BMI, maternal weight and/or weight gain and/or height), that provided statistical tests such as odds ratio (OR) or relative risk (RR) and on LBW, PTB or IUGR. The search provided 310 records, and 11 referred to the context in developing countries (as defined by the World Bank (http://data.worldbank.org/about/country-classifications/country-and-lending-groups; accessed 4 October 2012)). Additionally, four more relevant studies were found when reference lists of selected studies were examined.

## Results

***MUAC***

Table 1 shows an overview of cut-off values for MUAC in developing countries for LBW, IUGR and PTB. Most studies indicate a MUAC ranging from <22.0 cm to <27.6 cm with statistical significance for LBW. Cut-off values of <22 and <23 cm were strongly indicative for identifying a PW as high risk for LBW. Cut-off values were not strongly linked to gestational age. As there are insufficient data available on IUGR and PTB, these outcomes were not further analysed.

**Table 1. Maternal Mid-Upper Arm Circumference:** *ORs measured against the reference MUAC <24 cm (implying that MUAC ≥24 cm is protective against LBW but that MUAC >27cm is statistically significant with regard to low risk to LBW); ^$^no p-value given; according to researchers this value is best cut-off limit with highest sensitivity and specificity product. In BOLD statistically significant

MATERNAL MUAC										
Study	**Countries**	**Study population**	**Subjects (n)**	**Study type**	**Measured**	**Cut-off value**	**Stat.test**	**LBW**	**IUGR**	**Pre-term**
**Karim, Mascie-Taylor 1997 29**	Bangladesh	PW attending MCH clinics	251 women	prospective	3rd trimester	**<22 cm**	OR (95%CI)	**3.36 (1.68-6.79)**		
					3rd trimester	**<23 cm**	OR (95%CI)	**5.01 (1.42-17.89)**		
					3rd trimester	**<24 cm**	OR (95%CI)	**2.91 (1.31-6.61)**		
**Verhoeff, Brabin, van Buuren et al 2001 30**	Malawi	PW attending antenatal services	1423 women	prospective; univariate analysis	at 1st antenatal visit (any time during gestation)	**<23 cm**	OR (95% CI)		**1.5 (1.1-1.9)**	**1.8 (1.3-2.3)**
				prospective; multivariate analysis	at 1st antenatal visit (any time during gestation)	**<23 cm**	OR (p-value)			**1.8 (p<0.003)**
**Mohanty, Prasad, Reddy et al 2006 3**	India	PW from antenatal clinics	395 women	prospective	1st trimester	**≤22.5 cm**	RR	**1.67^$^**		
**Ogbanna, Woelk, Ning et al 2007 31**	Zimbabwe	PW admitted in hospital for labour and delivery	498 women	cross-sectional	end of pregnancy	24 cm	OR (95%CI)	0.54 (0.26-1.13)*		
					end of pregnancy	25-26 cm	OR (95%CI)	0.38 (0.18-0.81)*		
					end of pregnancy	**>27 cm**	OR (95%CI)	0.40 (0.19-0.84)*		
**Rollins, Coovadia, Bland 2007 32**	South Africa	PW attending antenatal services	2529 women	prospective	unclear, possibly at delivery	**<27.6 cm**	OR (p-value)	**1.77 (p<0.001)**		
**Ojha and Malla 2007 33**	Nepal	PW delivering in a hospital	308 women	prospective	delivery	**<22 cm**	OR (95%CI)	**2.04 (1.14-3.63)**		
**Dhar, Bhadra 2008 34**	Bangladesh	PW attending hospital	316 women	cross-sectional	during pregnancy	<22 cm	OR (95%CI)	1.26 (0.47-3.24)		
					during pregnancy	<24 cm	OR (95%CI)	1.71 (0.89-3.32)		
					during pregnancy	<26 cm	OR (95%CI)	1.68 (0.89-3.52)		
**Elshibly, Schmalisch 2008 35**	Sudan	PW delivering in a hospital	1000 women	prospective	delivery	<27 cm	RR (95%CI)	1.02 (0.63-1.65)		
**Sen, Roy, Mondal 2010 36**	India	PW delivering in a hospital	503 women	cross-sectional	delivery	**<22.0 cm**	RR (p-value)	**3.6 (p<0.0001)**		
**Sebayang, Dibley, Kelly et al 2012 37**	Indonesia	PW part of SUMMIT trial	14040 births	prospective	during pregnancy	**<23 cm**	OR (95%CI)	**1.16 (1.06-1.27)**		**1.47 (1.31-1.65)**
**Assefa, Berhane, Worku 2012 38**	Ethiopia	PW attending health services	956 women	prospective	during pregnancy	**<23 cm**	OR (95%CI)	**1.6 (1.19-2.19)**		

***BMI***

Table 2 shows an overview of cut-off values for BMI in developing countries for LBW, IUGR and PTB. Most studies indicate a BMI ranging from <18.5 kg/m2 to <20.5 kg/m2 with statistical significance for LBW. BMI changes during pregnancy, and there is insufficient evidence from this to indicate one cut-off value for a specific gestational age for BMI in developing countries. As there are insufficient data available on IUGR and PTB, these were not further analysed.

**Table 2. Maternal Body Mass Index:** *ORs measured against the reference BMI <22.8 kg/m2 (implying that BMI ≥22.8 kg/m2 is protective against LBW but that BMI >27.1 kg/m2 is statistically significant with regard to low risk to LBW); ^$^no p-value given; according to researchers this value is best cut-off limit with highest sensitivity and specificity product. In BOLD statistically significant

MATERNAL BMI										
**Study**	Countries	Study population	Subjects (n)	Study type	Measured at	Cut-off value	Stat.test	LBW	IUGR	Pre-term
**Karim, Mascie-Taylor 1997 29**	Bangladesh	PW attending MCH clinics	251 women	prospective	3rd trimester	**<18.5 kg/m2**	OR (95%CI)	**7.6 (1.89-32.54)**		
					3rd trimester	**<20.5 kg/m2**	OR (95%CI)	**6.47 (3.15-13.37)**		
					3rd trimester	**<22.5 kg/m2**	OR (95%CI)	**3.32 (1.53-7.31)**		
**Mohanty, Prasad, Reddy et al 2006^3^**	India	PW from antenatal clinics	395 women	prospective	1st trimester	**≤20.0 kg/m2 **	RR	**2.16^$^**		
**Sahu, Agarwal, Das et al 2007^39^**	India	PW delivering in a hospital	380 women	prospective	early second trimester	**<19.8 kg/m2**	RR (95%CI)	**2.1 (1.2-3.7)**	1.3 (0.5-3.6)	0.6 (0.1-3.9)
**Ogbanna, Woelk, Ning et all 2007^31^**	Zimbabwe	PW admitted in hospital for labour and delivery	498 women	cross-sectional	end of pregnancy	22.8-24.6 kg/m2	OR (95%CI)	0.51 (0.25-1.01)*		
					end of pregnancy	24.6-27.1 kg/m2	OR (95%CI)	0.51 (0.26-1.02)*		
					end of pregnancy	**>27.1 kg/m2**	OR (95%CI)	0.25 (0.10-0.60)*		
**Ojha and Malla 2007^33^**	Nepal	PW delivering in a hospital	308 women	prospective	measured at delivery	<18.5 kg/m2	OR (95%CI)	1.9 (0.61-5.65)		
**Elshibly, Schmalisch 2008^35^**	Sudan	PW delivering in a hospital	1000 women	prospective	delivery	<25 kg/m2	RR (95%CI)	1.15 (0.81-1.62)		
**Sen, Roy, Mondal 2010^36^**	India	PW delivering in a hospital	503 women	cross-sectional	delivery	**<18.5 kg/m2**	RR (p-value)	**2.9 (p<0.0001)**		

***Maternal weight for gestational age***

Table 3 shows an overview of cut-off values for maternal weight for gestational age in developing countries for LBW, IUGR and PTB. Most studies indicate a maternal weight for gestational age ranging from <43.5 kg to <50 kg with statistical significance for LBW. There is no clear cut-off value for maternal weight per gestational age, but <45 kg seems indicative for high risk of LBW in Asian countries regardless gestational age. As there are insufficient data available on IUGR and PTB, these were not further analysed.

**Table 3. Maternal weight for gestational age:** ^$^no p-value given; according to researchers this value is best cut-off limit with highest sensitivity and specificity product. In BOLD statistically significant

**MATERNAL WEIGHT FOR GESTATIONAL AGE**										
**Study**	Countries	Study population	Subjects (n)	Study type	Measured at	Cut-off value	Stat.test	LBW	IUGR	Pre-term
**Karim, Mascie-Taylor 1997^29^**	Bangladesh	PW attending MCH clinics	251 women	prospective	3rd trimester	**<43.5 kg**	OR (95%CI)	**12.27 (4.74-32.48)**		
					3rd trimester	**<45 kg**	OR (95%CI)	**8.47 (3.71-19.58)**		
					3rd trimester	**<50 kg**	OR (95%CI)	**4.58 (2.25-9.40)**		
**Mohanty, Prasad, Reddy et al 2006^3^**	India	PW from antenatal clinics	395 women	prospective	1st trimester	**≤45 kg**	RR	**2.28^$^**		
**Bisai, Mahalanabis, Sen et al 2007^40^**	India	PW attending obstetric ward	295 women	retrospective, cross-sectional	early second trimester (weeks 14-18)	**<45 kg**	OR (95%CI)	**2.06 (1.22-3.48)**	**3.06 (1.32-7.25)**	1.48 (0.63-3.48)
**Ojha and Malla 2007^33^**	Nepal	PW delivering in a hospital	308 women	prospective	delivery	**<45 kg**	OR (95%CI)	**3.5 (1.82-6.77)**		
**Elshibly, Schmalisch 2008^35^**	Sudan	PW delivering in a hospital	1000 women	prospective	delivery	<66 kg	RR (95%CI)	1.21 (0.87-1.7)		
**Bisai, Datta, Bose etc al 2009^41^**	India	PW coming for antenatal check up	233 women	retrospective, cross-sectional	24-28 weeks	**≤48 kg**	OR (95%CI)	**2.92 (1.56-5.51)**		

***Maternal weight gain***

There were insufficient data available demonstrating OR or RR on overall weight gain and cut-off values in developing countries for PW in relation to LBW, IUGR and PTB.

***Maternal height***

Table 4 shows an overview of cut-off values for maternal height in developing countries for LBW, IUGR and PTB. Most studies indicate a maternal height ranging from <146 cm to <156 cm with statistical significance for LBW. There is no clear one cut-off value for maternal height. As there were insufficient data available on IUGR and PTB, these were not further analysed.

**Table 4. Maternal height:** ^$^no p-value given; according to researchers this value is best cut-off limit with highest sensitivity and specificity product. In BOLD statistically significant

**MATERNAL HEIGHT**										
**Study**	Countries	Study population	Subjects (n)	Study type	Measured at	Cut-off value	Stat.test	LBW	IUGR	Pre-term
**Verhoeff, Brabin, van Buuren et al 2001^30^**	Malawi	PW attending antenatal services	1423 women	prospective; univariate analysis	at 1st antenatal visit (any time during gestation)	<150 cm	OR (95% CI)		**1.5 (1.2-2.0)**	**1.5 (1.1-2.0)**
				prospective; multivariate analysis	at 1st antenatal visit (any time during gestation)	<150 cm	OR (p-value)		**1.6 (p<0.003)**	
**Mohanty, Prasad, Reddy et al 2006^3^**	India	PW from antenatal clinics	395 women	prospective	1st trimester	≤152 cm	RR	**2.08^$^**		
**Ojha and Malla 2007^33^**	Nepal	PW delivering in a hospital	308 women	prospective	delivery	<145 cm	OR (95%CI)	1.87 (0.98-5.65)		
**Dhar, Bhadra 2008^34^**	Bangladesh	PW attending hospital	316 women	cross-sectional	during pregnancy	<146 cm	OR (95%CI)	**3.1 (1.37-6.95)**		
					during pregnancy	<151 cm		**2.66 (1.3-5.49)**		
					during pregnancy	<156 cm		1.21 (0.50-3.02)		
**Elshibly, Schmalisch 2008^35^**	Sudan	PW delivering in a hospital	1000 women	prospective	delivery	<156 cm	RR (95%CI)	**1.52 (1.05-2.2)**		

## Discussion

This study had several limitations. The literature review examined articles published in English. Though most studies only examined adult pregnant women, some also included pregnant adolescents who may have differences in physiology and anthropometry compared with their adult peers. Studies varied in sample size, methodology and context, and therefore comparisons should be done with care. The literature on humanitarian contexts and anthropometry in PW is limited in peer reviewed journals. There is likely more data in the grey literature, but as these are not peer-reviewed, they were not included. Maternal outcomes, especially maternal mortality, are of crucial importance to the foetus and infant. However, very few studies have occurred after the WHO Collaborative Study from 1995 to link maternal anthropometry during pregnancy and maternal survival. Only one study, a large prospective study in Nepal amongst almost 26,000 pregnancies, demonstrated that a MUAC of approximately 21-22 cm increased risk of maternal mortality24.

All examined anthropometric indicators represent some form of presently existing malnutrition in a PW, except for maternal height which represents malnutrition in the past. The best anthropometric indicator to use in a humanitarian context would be a measurement that is simple, easy to conduct, and ideally unrelated to gestational age as the latter is generally not exactly known in the contexts where humanitarian emergencies take place. An added value would be that the indicator can be ‘universally’ used, especially, for African or Asian contexts where many humanitarian emergencies occur.

**BMI**

BMI has been shown to reflect body composition of PW; lower BMI relates to wasting of both fat and lean tissue 13 ^,^ 15. BMI is a composite indicator that needs two measurements (weight, height) and skilled staff to calculate it. In a recently published large meta-analysis it was found that PW with a BMI ranging from ≤18.3 to ≤23 kg/m2 (but <20 kg/m2 in most studies) increased the risk of having an LBW infant (RR 1.52, 95%CI: 1.25–1.85) in developing countries 16. BMI can vary substantially during pregnancy, but it is an indicator of risk for LBW. However, no narrow range of cut-off points exist that can be used for a specific trimester in the Asian or African context. More studies need to be undertaken to research specific cut-off points for BMI to be measured, for example, in the first, second or third trimester, and that can identify risk for PW on LBW.

***Maternal weight for gestational age***

The WHO Collaborative Study from 1995 comprised 10 countries (predominantly developing countries). It concluded that a single measurement of attained maternal weight at 16-20 weeks (month 5) or 24-28 weeks (month 7) was the most practical screening instrument for LBW in most primary health care settings and provided an indication for intervention. Cut-off values for month 5 ranged from 40-53.5 kg (OR 2.4, 95%CI: 2.0-2.8) and for month 7 from 42.5-57 kg (OR 2.4, 95%CI: 2.1-2.7) 14 ^,^ 17. The WHO study provided a wide range in maternal weight for gestational age. In the humanitarian context where gestational age is often not exactly known, it is not possible to recommend a cut-off value on maternal weight per gestational age for universal use. It would be worthwhile to further investigate if <45 kg at any time of pregnancy could be used in future emergencies in the Asian context.

***Maternal weight gain***

The WHO Collaborative Study 1995 showed that weekly weight gain varying from 50-300 g between months 5 and 7 or months 5 and 9 were indicative of LBW (OR 1.6 (95%CI: 1.3-2.0) and 1.7 (95%CI: 1.3-2.2) respectively)^14^. A large systematic review of outcomes of maternal weight gain found strong evidence to support the association between low gestational weight gain and LBW 18. Han et al showed in another large meta-analysis that low total gestational weight gain (defined as <11.5 to <12.5 kg for normal or underweight women, respectively) was also associated with increased risks of LBW in developing countries (RR 1.84 (95%CI: 1.71–1.99)) 19. This would indicate that, on average, weekly weight gain of <300 g would indicate high risk. However, the same meta-analysis also showed that low weekly gestational weight gain had a significant effect on PTB but not on LBW 19.

As there is no clear evidence of which weight gain cut-off is most sensitive to LBW, and as weight gain changes per trimester and a minimal of two measurements are needed, this indicator is not be the useful for screening purposes in emergencies.

***Maternal Height***

The WHO Collaborative Study 1995 showed a similar range as noted in Table 4 of 146-157 cm (OR 1.7, 95%CI: 1.6-1.8) when highest and lowest quartiles of the maternal height distribution were compared 14. Maternal height as potential indicator for LBW lacks a clear cut-off value for general use in developing countries to identify LBW risk. However, short maternal stature is strongly associated with an increased risk of obstructed labour due to cephalo-pelvic disproportion 20 ^-^ 23 and infant underweight 22. Thus, short maternal stature (146-157 cm) and can be used as such to identify risk for LBW; furthermore it can be used to identify women with obstetric risks.

**MUAC**

MUAC is a good indicator of the protein reserves of a body, and a thinner arm reflects wasted lean mass, i.e. malnutrition 25. The WHO Collaborative Study 1995 showed MUAC cut-off values of <21 to 23 cm as having significant risk for LBW (OR 1.9, 95%CI: 95% 1.7-2.1) when highest and lowest quartiles of the maternal height distribution were compared 14. These values are similar to values identified in this review. However, the data in Table 1 indicate that a cut-off value of 21 cm might be too low. As LBW has detrimental effects on a child’s survival it seems that a more inclusive approach with a MUAC cut-off of <22 or <23 cm should be used to indicate risk of LBW and to use as entry criterion for nutritional programmes. MUAC is rather insensitive to changes over the total period of pregnancy for adult women 3 ^,^ 26 ^-^ 28, is easy to measure, and requires only one measurement. More research is needed whether different cut-off values should be used for the Asian or African continent, but current data suggest that <23 cm appears adequate for both continents. It is also the most conservative cut-off value ensuring the most PW at risk for LBW are included. It is likely that the relevance of the use of MUAC is similar in different humanitarian emergencies, being it conflict, natural disaster, sudden or slow onset.

Currently, there is no data available that differentiates PW from being moderately or severely malnourished, i.e. having categories for MUAC that indicate high or relatively even higher risks for adverse outcomes. This does not mean they do not exist, but that this literature review does not provide sufficient evidence to support the creation of such categories.

Further research is needed to evaluate whether the combined use of one or two easily measurable anthropometric indicators can have a high predictive power for risk of adverse birth outcomes in humanitarian contexts. In addition, research is needed to determine to what extent enrolment in nutritional programmes of PW with a MUAC <23 cm can avert risk of LBW.

## Conclusions

In the humanitarian context, MUAC can be used as a reliable indicator of risk of LBW. A cut-off value of <23 cm should be used to enrol PW in nutritional programmes. National protocols from Ministries of Health and humanitarian organisations that currently use a MUAC <21 cm to enrol PW in SFPs should consider increasing the cut-off value in order to reduce the risk of LBW infants.

## Competing interests

The authors have declared that no competing interests exist.

## Correspondence

Mija-tesse Ververs; Email: mijaververs@hotmail.com
